# Evaluation of starch granules based on hydroxypropylcellulose as a substitute for excipient lactose

**DOI:** 10.1186/s40780-024-00354-w

**Published:** 2024-06-21

**Authors:** Tomohiro Yoshikawa, Hiroyo Okamoto, Kenta Takeuchi, Atsushi Hirata, Hiroko Otake, Noriaki Nagai

**Affiliations:** 1 Kindai University Nara Hospital, 1248-1 Otodacho, Ikoma, Nara, 630-0293 Japan; 2 Faculty of Pharmacy, Kindai University, 3-4-1 Kowakae, Higashi-Osaka, Osaka 577-8502 Japan

**Keywords:** Starch granules, Hydroxypropylcellulose, Binding agent, Excipient; lactose, Particle size

## Abstract

**Background:**

The improvement in flowability and adhesion of starch powder (SP) is essential for using starch as an excipient for lactose intolerant patients. In this study, we attempted to evaluate the usefulness of hydroxypropylcellulose with molecular weight 80,000 (HPC-80) in the preparation of the starch granules (SG) as a substitute for excipient lactose.

**Methods:**

Hydroxypropylcellulose with molecular weight 30,000 (HPC-30) and HPC-80 were used as binders to prepare the SG, and defined as HPC-30-SG and HPC-80-SG, respectively. Mean particle size (D50) was measured according to the Method, Optical Microscopy of Particle Size Determination in Japanese Pharmacopoeia, Eighteenth Edition, and storage stability were evaluated by measuring of the physical properties after vortexing the granules for 180 s (physical impact). The product loss rate was calculated from the weight change of the various excipients before and after the one dose packaging (ODP).

**Results:**

The D50 of SP (30 µm) was smaller than that of the lactose powder (115 µm). The granulation with 0.75–3% HPC-30 and HPC-80 increased the particle size of SP, and the D50 in 1.5% HPC-30-SG (255 µm) and HPC-80-SG (220 µm) were higher than that of lactose. The excipient was removed from the heat seal of the ODP, and upon visual inspection, a large amount of starchy material was observed to be adhering to the paper in the SP. On the other hand, the low recovery rate in SP was attenuated by the granulation with HPC-30 and HPC-80. In the both HPC-30 and HPC-80, the improvement in recovery rate reached a plateau at 1.5%, and the levels of recovery rate was similar to that of lactose. The recovery rate in the 0.75–3% HPC-30-SG and 0.75% HPC-80-SG were decreased by the physical impact, however, the recovery rate and amount of 1.5% and 3% HPC-80-SG were not affected by the physical impact, and these levels were similar to that of lactose.

**Conclusions:**

The use of HPC-80 as a binder of SG was found to produce a higher quality granule product than conventional HPC-based SG. This finding is useful in streamlining the preparation of starch-based powdered medicine in clinical applications.

## Background

In pediatric pharmacotherapy, tablets and capsules are often difficult to swallow and dosages are commonly minimal; hence, excipients are frequently utilized to make drug-containing powdered medicine in hospitals and dispensing pharmacies. Lactose is the often used as excipient in these mixtures [1]; however, it is contraindicated in patients who are lactose intolerant. Lactose intolerance emanates because of various underlying causes, including being hereditary or secondary to infectious gastroenteritis [2, 3]. Moreover, apart from intolerance in certain patients, lactose is known to trigger complex chemical reactions with isoniazid and aminophylline [4]. Owing to this, substitution of lactose with starch powder (SP), mannitol or crystalline cellulose are recommended when use of lactose is contraindicated or not possible. However, the SP, one of the alternatives to lactose, adheres to drug packaging material, resulting in uneven distribution. In clinical practice, the absence of residual powder on the packaging improves therapeutic efficacy from adequate patient dosing. Therefore, addressing this issue is essential for consistent administration of the required dose and ensuring the accuracy of a single delivery.

Previous studies have shown that increasing the particle size of excipients improves powder flowability [5]. Furthermore, some studies have examined the improvement of flowability following the granulation of starch bases [5]. Hydroxypropylcellulose with molecular weight 30,000 (HPC-30) is a derivative of cellulose used as an additive (binder) in dosage forms. It is amphiphilic and is used as a binder or coating agent for pharmaceutical tablets. Hydroxypropylcellulose with molecular weight 80,000 (Klucel™ EXF Ultra hydroxypropylcellulose) (HPC-80) is a tablet binder that ensures formulation predictability, reliability, and robustness. HPC-80 provides enhanced tablet strength and low friability, even at low doses and with the most difficult to compress active pharmaceutical ingredients. Thus, we selected HPC-80 based on these characteristics, and HPC-80 may be useful as a new binder with a small particle size [5], and is expected to yield good results during drug or dosage form formulation. Herein, we compared the improvement in flowability and recovery of starch granules (SG) using two different binders (HPC-30 and HPC-80).

## Methods

### Preparation of SG

The 0.75%, 1.5%, and 3% binding agents (HPC-30 or HPC-80) were dissolved in 200 mL anhydrous ethanol, and 500 g of SP was added to the ethanol solution with the binding agent and stirred. Following this, the mixture was granulated using a sieve (mesh size 1.40 mm, wire diameter 0.71 mm), allowed to dry naturally for 24 h, and then sieved through a other sieve (mesh opening 355 μm, wire diameter—244 μm). The resultant SG from HPC-30 or HPC-80 were denoted HPC-30-starch granules (SG) or HPC-80-SG, respectively. The selection of binding agents was based on those that are commonly used and generally regarded as safe [5, 6]. HPC-80 (molecular weight 80,000) was selected to investigate whether the HPC-80, which has a higher molecular weight than HPC-30 (molecular weight 30,000) is suitable as binders to prepare the SG in this study. In this study, HPC-80 (Klucel™ EXF Ultra hydroxypropylcellulose, Lot number: 190455 and 190,003) was obtained from Ashland Inc., US-KY, and the HPC-30 (Lot number: WDP4322 and PAQ1730) was provided from FUJIFILM Wako Pure Chemical Corporation (Osaka Japan). Starch powder (potato starch, Lot number; OH02) and lactose (lactose monohydrate, Lot number; M019BF4) were purchased from Viatris Global Healthcare Company (Tokyo Japan) and KENEI Pharmaceutical Co. (Osaka Japan), respectively. The HPC-30 (Lot number: PAQ1730) and HPC-80 (Lot number 190003) were used to measure particle size distribution and recovery rate in lot-to-lot (lot-to-lot reproducibility).

### Measurement of particle size

Particle size was measured according to the Method, Optical Microscopy of Particle Size Determination in Japanese Pharmacopoeia, Eighteenth Edition (feret's diameter) [7]. Briefly, the granules were observed using a phase contrast microscope (Olympus Corporation, Tokyo Japan), and the particle size in the images was determined using ImageJ v1.52 software (NIH US-MD). The mean particle size (D50) was evaluated by expressing the particle size distribution as a cumulative distribution and creating a linear fitting with a function in an Excel spreadsheet (*n* = 200) [8].

## Adhesion of SGs during one dose packaging

A fully automated packaging machine YS-93SRzII (Yuyama Manufacturing Co., Ltd., Osaka, Japan) was used for one dose packaging (ODP). The lactose, SP, and various SGs were dispensed at a rate of 0.2 g/package [9], and the speed of the fully automated packaging machine was set to 40 packets/min. The adhesion of SGs during the ODP process was determined by comparing the weight before and after dispensing, and recovery rate (%) was evaluated following equation; (weight without dispensing – weight with dispensing)/weight without dispensing × 100.

## Evaluation of stability against physical impact

The various SGs were added to a plastic bottle, and vortexed for 180 s using a vortexer (AS ONE Corporation, Osaka Japan). Thereafter, the particle size and adhesion during the ODP process (recovery rate) were evaluated for any changes as described above.

## Results

**Fig. 1:**
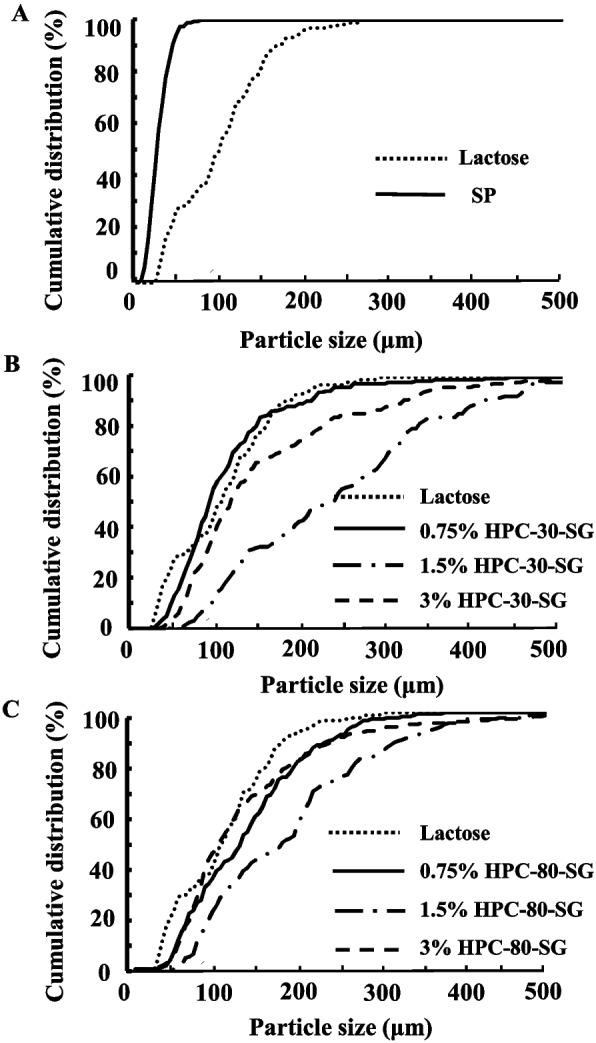
Particle size distribution of SGs with or without binding agent. **A** Particle size distribution of lactose and SP, **B** Particle size distribution of 0.75%–3% HPC-30-SG; **C** Particle size distribution of 0.75%–3% HPC-80-SG

Figure 1 and Table 1 show the particle size distribution (Fig. 1) and D50 (Table 1) of the various SGs. The particle size of SP was smaller than that of the lactose powder. The granulation with binding agent (HPC-30 and HPC-80) increased the particle size of the SP, and the median diameter of SGs ranged from 100–255 μm. In the both HPC-30-SG and HPC-80-SG, the particle size was largest at 1.5%, and the particle size at 0.75% and 3% were similar.

**Table 1:** Effect of physical impact on the D50 and adhesion of excipients to heat seal for ODP

Excipient		D50 (µm)		Recovery amount (mg)	
		Before stirring	After stirring	Before stirring	After stirring
Lactose		115 ± 0.063	107 ± 0.059	192 ± 0.0104	190 ± 0.0108
SP		30 ± 0.015^*^	29 ± 0.013^*^	137 ± 0.0299^*^	141 ± 0.0278^*^
HPC-30-SG	0.75%	100 ± 0.081^*^	70 ± 0.045^*^	165 ± 0.0182^*^	163 ± 0.0179^*^
	1.5%	255 ± 0.133^*^	165 ± 0.010^*^	188 ± 0.0077	187 ± 0.0076
	3%	130 ± 0.113^*^	110 ± 0.084	190 ± 0.0102	189 ± 0.0101
HPC-80-SG	0.75%	140 ± 0.076^*^	85 ± 0.041^*^	156 ± 0.0166^*^	153 ± 0.0163^*^
	1.5%	220 ± 0.122^*^	195 ± 0.101^*^	190 ± 0.0093	191 ± 0.0090
	3%	110 ± 0.108^*^	95 ± 0.052^*^	192 ± 0.0081	191 ± 0.0082

Mean ± S.D. *n* = 4 (D50) and 30 (recovery amount). *P < 0.05 vs. Lactose for each category (Tukey–Kramer test)

**Fig. 2:**
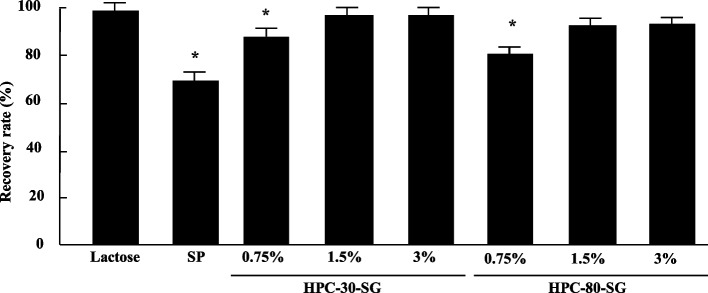
Recovery rate of SGs with or without binding agent during the ODP heat seal process. Mean ± standard deviation (S.D.). *n* = 30. *P < 0.05 vs. Lactose for each category (Tukey–Kramer test)

Figure 2 shows the recovery rate, during heat sealing of the ODP process of various SGs formulated using HPC-30 and HPC-80. The low recovery rate in SP was attenuated by the granulation with HPC-30 and HPC-80, and the recovery of 1.5% and 3% of HPC-30-SG and HPC-80-SG were significantly higher than that of SP. In the both HPC-30 and HPC-80, the improvement in recovery rate reached a plateau at 1.5%, and the levels of recovery rate was similar to that of lactose.

**Fig. 3:**
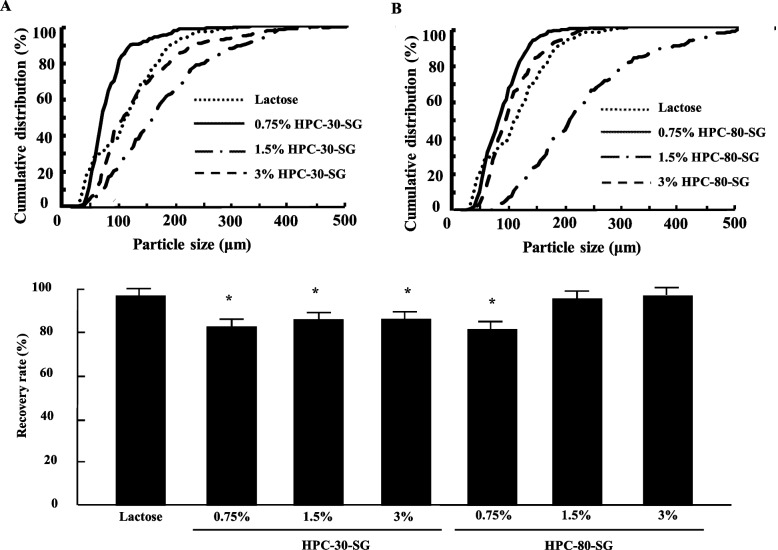
Changes in particle size and recovery rate of various SGs after physical impact. **A** Particle size distribution of SGs with 0.75%–3% HPC-30-SG treated with physical impact; **B** Particle size distribution of SGs with 0.75%–3% HPC-80-SG treated with physical impact. **C** Effect of HPC-30 and HPC-80 content on recovery rate of SGs exposed to physical impact. Mean ± S.D. *n* = 30. *P < 0.05 vs. Lactose for each category (Tukey–Kramer test)

Figure 3A and B shows the changes in particle size of HPC-30-SG and HPC-80-SG following physical impact. The particle size of prepared SGs in this study were decreased by the physical impact. The particle size in 0.75% HPC-30-SG was lower than that of lactose. Contrast with the result of 0.75% HPC-30-SG, the particle size in 3% HPC-30-SG and 0.75% and 3% HPC-80-SG treated with physical impact were similar to that of lactose. On the other hand, the particle size in 1.5% HPC-30-SG and HPC-80-SG treated with physical impact were larger than that of lactose. Figure 3 and Table 1 show the effect of physical impact on the recovery rate and amount of HPC-30-SG and HPC-80-SG during ODP heat sealing. The recovery rate and amount in the HPC-30-SG treated with physical impact were lower than that of lactose, the level was approximately 83% regardless of the HPC-30 contents. The recovery rate and amount in the 0.75% HPC-80-SG were also decreased by the physical impact, however, the recovery rate and amount of 1.5% and 3% HPC-80-SG were not affected by the physical impact, and these levels were similar to that of lactose. Moreover, the packaging error in 1.5% and 3% HPC-80-SG tend to be lower in comparison with other SP and corresponding SGs (Table 1). In addition, we measured the particle size distribution and recovery rate in lot-to-lot of the corresponding 1.5% HPC-30-SG and HPC-80-SG. The D50 without stirring of HPC-30-SG and HPC-80-SG were 254 ± 0.12 µm, 219 ± 0.116 µm, and the D50 with stirring of HPC-30-SG and HPC-80-SG were 163 ± 0.009 µm and 198 ± 0.095 µm, respectively (*n* = 4). Moreover, the recovery amount without stirring of HPC-30-SG and HPC-80-SG were 187 ± 0.0083 mg, 189 ± 0.0086 mg, and the recovery amount with stirring of HPC-30-SG, HPC-80-SG were 185 ± 0.0090 mg and 190 ± 0.0094 mg, respectively (*n* = 30).

## Discussion

First, we measured the particle size of excipients as powder, since the particle size was related the flowability (Fig. 1). The particle size of SP was smaller than that of the lactose. This result suggested that this implies that the flowability of starch is low and should be improved as it would likely cause packaging errors and result in residual powder on packaging paper. Subsequently, we attempted to prepare the SGs, and commercial HPC-30 and HPC-80 of different molecular weights were used as binders in the process, since the granule can be not provided by the granulation without binding agent (Fig. 1). Granulation increased the particle size of the SP, and the particle size of the HPC-30-SG and HPC-80-SG were larger than that of lactose (Fig. 1). The content of binder is also important in the production of SGs, since the binder strongly agglomerates between particles. In addition, it was known that the granule stability decreases when the amount added is too large [10], and an optimal concentration is required to prepare SGs. We showed that the 4.5% concentration was determined to be too viscous for use following preliminary investigations. Therefore, 0.75%, 1.5%, and 3% binder concentrations were used in this study. On the other hand, the particle size of the HPC-30-SG and HPC-80-SG was difference, and the largest particle size of granules at a 1.5% addition rate. The molecular weights of HPC-80 and HPC-30 are approximately 80,000 and 30,000, respectively, and the viscosity of HPC-80 is higher than that of HPC-30 (the viscosity of 1.5% HPC-30 and HPC-80 in water are 5 mPa·s, 18 mPa·s, respectively, 25℃). Taken together, these differences in the degree of polymerization, molecular weight, and viscosity may affect the particle size of SGs in this study.

Next, we measured the adhesion and recovery rate, respectively, during heat sealing of the ODP process of HPC-30-SG and HPC-80-SG (Fig. 2). The recovery of HPC-30-SG and HPC-80-SG was significantly higher than that of the SP, although, the recovery of both 0.75% HPC-30-SG and HPC-80-SG were significantly lower in comparison with 1.5% and 3% HPC-30-SG and HPC-80-SG. Moreover, the recoveries of 1.5% and 3% of HPC-30-SG were comparable to those of lactose. These results indicated that the SGs using HPC-30 and HPC-80 improved the issue observed with SP during ODP [11], given that the investigated excipients with binding agents are not inferior to lactose. On the other hand, it is important to clarify the lot-to-lot reproducibility. Therefore, we measured the particle size distribution and recovery rate in lot-to-lot of the corresponding 1.5% HPC-30-SG and HPC-80-SG (lot-to-lot reproducibility), and the no lot-to-lot differences were observed.

Moreover, evaluation of granule durability is of practical importance. Therefore, we demonstrated the changes in storage and transport properties of SGs when they were subjected to physical impact (Fig. 3). Following physical impact, 1.5% and 3% HPC-80-SG were not inferior to lactose, while other SGs were inferior with significant differences. From this result, 1.5% and 3% HPC-80-SG can be considered appropriate excipients to substitute lactose. In addition, the packaging error in 1.5% and 3% HPC-80-SG tend to be lower in comparison with other SP and corresponding SGs (Table 1). These results suggested that 1.5% and 3% HPC-80-SG provided fewer mistakes during packaging result in an accurate dose.

The results of this basic experiment demonstrated the formulation's usefulness, and the possible advantages of using this formulation in the clinical setting are following; (I) hospital preparation allows for tailoring prescriptions to meet individual patient needs or specific cases, providing optimal treatment. (II) it enables tighter quality control, especially when specific requirements for preparation and compounding are necessary. (III) a smaller scale can reduce formulation costs compared to mass production. On the contrary, demerits is following; (I) it is time-consuming and labor-intensive. Individual compounding for each prescription may restrict time available for other tasks or patient care. (II) it is a higher risk of errors or variability, Errors could affect efficacy or safety, requiring careful attention. These are the research limitations of this experiment, and it requires careful consideration and appropriate quality management.

In Japan, commercially available corn starch granules is able to provide from Japan corn starch co., ltd. (Japan). However, the D50 is 81.58 µm, and larger particle size is needed to attenuate the adhesion of corn starch granules during one dose packaging. In fact, the recovery rate of 0.75% HPC-30-SG, which is comparable particle size, is not enough (Figs. 2 and 3). Therefore, the preparation of SG using binding agents (HPC-30 or HPC-80) would be useful. On the other hand, it is possible to show similar effects with other HPC-30 derivatives by setting optimal concentrations. Further studies are needed to determine the optimal concentration settings for other HPC-30 derivatives. In conclusion, we designed SGs using HPC-30 or HPC-80 as binding agents, and found that drug loss during the ODP process was improved when between 1.5%–3% HPC-80 was used.

## Data Availability

The datasets used and/or analyzed during the current study are available from the corresponding author on reasonable request.

## Abbreviations

HPC-30: Hydroxypropylcellulose with molecular weight 30,000
HPC-80: Hydroxypropylcellulose with molecular weight 80,000
ODP: One dose packaging
S.D.: Standard deviation
SG: Starch granule
SP: Starch powder
